# The Role of Remote Monitoring in Evaluating Fatigue in Multiple Sclerosis: A Review

**DOI:** 10.3389/fneur.2022.878313

**Published:** 2022-06-27

**Authors:** Valerie J. Block, Riley Bove, Bardia Nourbakhsh

**Affiliations:** ^1^Department of Neurology, Weill Institute for Neurosciences, University of California, San Francisco, San Francisco, CA, United States; ^2^Department of Neurology, Johns Hopkins University, Baltimore, MD, United States

**Keywords:** remote monitor, accelerometry, sensors, fatigue, fatigability, remote evaluation, multiple sclerosis

## Abstract

Fatigue is one of the most common multiple sclerosis (MS) symptoms. Despite this, monitoring and measuring fatigue (subjective lack of energy)– and fatigability (objectively measurable and quantifiable performance decline)– in people with MS have remained challenging. Traditionally, administration of self-report questionnaires during in-person visits has been used to measure fatigue. However, remote measurement and monitoring of fatigue and fatigability have become feasible in the past decade. Traditional questionnaires can be administered through the web in any setting. The ubiquitous availability of smartphones allows for momentary and frequent measurement of MS fatigue in the ecological home-setting. This approach reduces the recall bias inherent in many traditional questionnaires and demonstrates the fluctuation of fatigue that cannot be captured by standard measures. Wearable devices can assess patients' fatigability and activity levels, often influenced by the severity of subjective fatigue. Remote monitoring of fatigue, fatigability, and activity in real-world situations can facilitate quantifying symptom-severity in clinical and research settings. Combining remote measures of fatigue as well as objective fatigability in a single construct, composite score, may provide a more comprehensive outcome. The more granular data obtained through remote monitoring techniques may also help with the development of interventions aimed at improving fatigue and lowering the burden of this disabling symptom.

## Introduction

### Evaluating Fatigue or Fatigability?

One of the more challenging aspects of research in multiple sclerosis (MS) fatigue is a lack of consensus on how to define, and therefore measure, this heterogeneous symptom.

Fatigue has variably been described as “an overwhelming sense of tiredness that is out of proportion to the performed activity” (1), “a feeling of difficulty initiating, or sustaining voluntary effort” (2), or “a feeling related to a lack of motivation to deploy resources” (3). A panel of experts (the MS Council for Clinical Practice Guidelines) defined MS **fatigue** as “a ***subjective*** lack of physical and/or mental energy that the individual or caregiver perceives to interfere with usual activities” (4). This definition not only points to the multidimensionality of MS fatigue and its negative impact on patient's life, but also emphasizes the subjective nature of this symptom.

However, this expert definition is still vague and does not answer many important and practical questions about the severity, temporality, or triggers of fatigue. For example, one patient may feel they do not have enough energy for going on a hike, and another may feel they do not have the energy to go from the living room to the mailbox. The severity, and perhaps, the “quality” of fatigue is very different between these two patients, yet the definition does not distinguish between the two, nor does it clarify if the subjective lack of energy happens before or after an effortful activity.

In contrast to the subjective feeling of lack of energy (fatigue), **fatigability** has been defined as a more ***objectively*** measurable and quantifiable performance decline in physical or cognitive tasks. Unfortunately, even the association between subjective fatigue and objective fatigability in MS is not straightforward, as noted in people with advanced MS where change in subjective fatigue did not correlate with cognitive fatigability (5). More encouragingly, subjective fatigue (measured with a validated questionnaire) was associated with an objective measure of physical activity (step count from an accelerometer, as a proxy for physical fatigability) in a cohort of MS with a wide range of disability scores (6). After exertion, a 6-min walk test, gait and motor parameters (postural sway, arm-swing and hand grip strength) demonstrated potential associations with fatigue ratings and fatigability scores (7). These emphasize the need for objective, validated measures that are able to capture real-time fatigability in people with MS (PwMS), during all moments of the day (i.e., during and after going for a hike, going to the mailbox, or sitting watching TV) and over many days at a time.

## Current Methods Of Evaluation For Fatigue and Fatigability and Their Limitations

### Fatigue

Clinical methods to characterize patients' feeling of fatigue use self-reported questionnaires (8). Data derived from self-report scales depend on the scale developer's conceptualization of fatigue and the respondent's interpretation of the questions (9). Some scales, developed to quantify fatigue in other medical conditions, are not specific to MS. Most fatigue questionnaires ask patients to retrospectively evaluate previous fatigue, and many have a look-back period of seven to 28 days (hence, calling these measurements **“trait” fatigue**) (10). However, the scores usually do not portray the average fatigue severity in the look-back period and are mainly influenced by the most recent and most severe fatigue states (11). These scales do not provide any information about either diurnal or day-to-day variations in fatigue severity, phenomena that are well-known to patients with MS and their clinicians (i.e. “having good days and bad days”) (10). The lack of granularity and placebo-responsiveness of fatigue measures from self-report questionnaires could represent significant limitations to identifying or developing effective fatigue treatments in MS (12).

To address the problem with recall bias, there has been an attempt to use self-report questions or questionnaires to assess the **fatigue “state”** (fatigue severity at the moment) (13). These include visual analog scales and/or asking patients to rate how severe their fatigue is at the moment of assessment. However, because of the diurnal and day-to-day variations of fatigue severity, “state” fatigue needs to be measured several times a day and over a longer epoch to provide a more comprehensive picture of a patient's fatigue severity. This in turn may increase the sense of fatigue in the patient.

### Fatigability

Considering the inherent limitations of self-report measures, efforts to measure fatigue more objectively have involved several physical and cognitive performance-based measures. In these tests, compared to healthy controls, patients with MS demonstrate a decline in physical (e.g., sustained muscle contraction) and cognitive function (e.g., visual and verbal memory) after an effortful continuous performance task (14). These declines can happen even if baseline muscle strength and cognitive performance are normal. To date, such objective declines in performance (which we defined as **fatigability**) may not correlate with self-reported fatigue (15, 16). This lack of correlation might be because self-reported fatigue has a look-back period and is supposed to measure “the average fatigue severity” over the look-back period, while the performance-based test measures the fatigability “at the moment.” This issue could be overcome by more frequent (or continuous) assessments of the performance.

The lack of correlation between subjective fatigue and objective fatigability may also be due to the multidimensionality of MS-related fatigue. In this case, it is important to incorporate both self-reported and performance-based measures when assessing fatigue in the research setting. Thus far, most clinical trials evaluating the efficacy of medication and interventions for MS-related fatigue have relied solely on self-reported questionnaires.

## Remote Evaluation of Fatigue and Fatigability

### Subjective Assessments: Fatigue

Almost all validated fatigue questionnaires can be administered and answered remotely by PwMS. These surveys can be accessed via a web page on patients' computers, smartphones or tables, from their homes or workplace. Remote evaluation of fatigue using patient surveys can obviate the need for a clinic visit and facilitates participation in fatigue research by reducing barriers (i.e., eliminating commutes to testing centers). Such a strategy was used in a clinical trial assessing the efficacy of pharmacotherapy for MS fatigue (12). The readability and acceptability of an electronic version of a recently-developed MS-specific fatigue questionnaire were formally demonstrated during the initial evaluation of the instrument (17). The advantage of computerized questionnaires also includes adaptive features, where the list of questions offered to a patient can change based on their answers to previous questions [e.g., Neuro-QOL fatigue survey (18)].

The ubiquitous availability and versatility of portable electronic devices and smartphones provide a unique opportunity to continuously obtain self-reported (fatigue; state and trait) and performance-based (fatigability) measures in patients' real-life settings. This methodology, referred to as ecological momentary assessment (EMA), involves the repeated sampling of subjects' experiences and behavior in the subjects' natural environment and in real-time (19). Applying the EMA to smartphones and electronic devices can create a set of observable behaviors from the interaction between human disease and the person's use of the technology, collectively referred to as digital phenotypes (20). In a study that used a handheld portable electronic device, a self-report of fatigue severity (by asking a single question) was prompted by auditory alarms multiple times a day. Fluctuation in fatigue in both PwMS and healthy individuals was demonstrated in this study (21). In another study, PwMS used a wrist-worn device to record Real-Time Digital Fatigue Scores (RDFS) several times a day, over 3 weeks. Mean RDFS correlated with traditional validated fatigue scores, and captured circadian variation in fatigue severity (22). In a similar way, smartphones can be used for gathering real-time, patient-reported fatigue severity several times a day and in various social situations. This eliminates the recall bias inherent to the currently used questionnaires. Smartphones can also be used to present patients with tasks (such as a reaction time task) to assess performance-based fatigue.

### Objective Assessments: Fatigability

In a disease as fluctuating as MS, where symptoms can change hourly, one-time clinic-based measures do not provide us with a complete picture of the persons' performance or deficits. Wearable technology has greatly enhanced the ability to monitor patients' function outside of the clinic; smaller and more discreet wearable monitors can be worn on various parts of the body to provide data from everyday life.

Changes in accelerometer or sensor-based gait and muscle activation metrics can be used to infer the users **fatigability** over minutes, hours or days (23, 24). Physical activity in PwMS is influenced by multiple factors, one of which is the patients current subjective energy levels (**state fatigue**) (25, 26). As noted, physical activity outcomes from accelerometry have been associated with conventional measures of perceived fatigue in MS (27–32). Self-reported fatigue (**state and trait**) has also been associated with sensor-based gait parameters, providing a more objective correlate to an otherwise subjective measure (7, 33–36).

#### Smartphones

Because texting and web browsing are among smartphones' most used features, keystroke dynamics (KD) data can be studied as a possible measure of fatigue in MS. KD is one of the behavioral biometric characteristics and is based on the assumption that different people have different typing manners. KD has constant and variable components. The constant component is dependent on the person's physical data and does not change over time. The variable component, however, is dependent on the person's psychological state. By associating changes in parameters such as typing speed, the number of mistakes, and usage of specific keys, changes in physical and mental behavior could be determined. For example, in a study of healthy subjects using specific key press and release timing information from text input tasks, average daytime fatigue recognition accuracy of 98% could be reached (37). Also, specific changes in smartphone usage and KD metadata were correlated with mood states in patients with bipolar affective disorder (38). Keystroke features differentiated between PwMS and healthy controls and were correlated with measures of disability, such as the Expanded Disability Status Scale (EDSS). However, KD data were not associated with traditional trait fatigue questionnaires, such as Fatigue Severity Scale (FSS) (39). This lack of correlation could be due to recall bias associated with traditional questionnaires. There is a possibility that KD data better reflect fatigability (as opposed to subjective fatigue). Future longitudinal studies with concurrent measurements of fatigue and fatigability can answer these questions.

#### Activity Monitors

##### Types of Activity Monitors

Many gait and activity assessment wearables exist, chiefly divided into *Activity* monitors: measuring the quantity of activity, and *Movement* monitors: for gait quality or movement. The pedometer is the simplest activity monitor - traditionally used to record step counts only (40). The most commonly used research devices in MS are triaxial waist-worn accelerometers (e.g., ActiGraphs) (41). However, these devices tend to express output in activity counts rather than step counts, which are potentially harder to interpret for the lay person. Some devices are designed to wear on lower limbs (i.e., ankle or thigh) (42). Despite variable correlation accuracy with manual step counts, they may not be practical for longer-term use as they look less like ‘trendy wearables' and more research or monitoring devices (43, 44). Other devices used in research adhere to the skin, for example, the ActivPAL or the BioStamp (45). A study in MS found that the BioStamp had high accuracy for detecting gait patterns and step number and perceived differences in gait characteristics by disability level (45). Inertial Measurement Unit (IMU) devices are also used for evaluation and monitoring. These small devices are comprised of accelerometers, gyroscopes, and magnetometers which measure linear acceleration, angular velocity, and magnetic field strength, respectively. They can be embedded in shoes or clothing, providing spatio-temporal data. Multisensors, using biaxial accelerometers with heat flux sensors, skin temperature sensors, near-body ambient sensors, galvanic skin response sensors are worn on an armband around the upper arm. These provide a comprehensive picture of activity as well as the environment and physiological state of the user at the time of data capture (46), and by measuring multiple elements are likely to be advantageous for the study of a heterogeneous, multidimensional symptom like fatigue.

#### Types of Monitoring Outcomes

Remote monitors generate an array of outcomes, including activity counts or step counts (using different levels of granularity and aggregated data summaries; daily or minute-by-minute, intensity, duration), gait kinematics (such as walking speed, stride length, width and cadence), energy expenditure, heart rate, breathing rate, burnt calories, sleep quality and duration, estimation of activity type, range of movement, distance traveled - and more. Due to the many factors and symptoms that can affect fatigue (state or trait) and fatigability, the use of remote wearable devices that can measure various outcomes concurrently in everyday life would be ideal. Supposedly due to restrictions in size and weight of the devices, none to date evaluate all outcomes in the home setting.

#### Real-World Examples

A significant benefit of wearable devices is their potential for *ecological and continuous* use. Therefore, commercially available devices made for ‘ease of use' and with fashion-conscious designs have made their way into clinical research to improve adherence in longitudinal studies. The Apple iWatch and Fitbit specifically have gained wide publicity (47–73).

In MS, studies evaluating physical activity using commercial wearables have shown (1) strong-moderate correlations between clinical and patient-reported disability measures (6, 74–80), (2) continuous observation provides less biased assessment vs. sporadic cross-sectional measures (6, 74, 81, 82), (3) fatigue is not the only factor affecting sedentary behavior and physical activity in MS (83, 84) and (4) that average daily step count (STEPS) is responsive to change over 1-year, even when conventional measures remain stable (74).

##### Associations With Fatigability (Performance) and Trait Fatigue (Patient Rating)

In the FITriMS study (a year-long observational study of continuous, remote ambulatory activity in PwMS) participants wore a Fitbit Flex for up to 2 years on their non-dominant wrist and were asked to complete online surveys every 6 months, including a subjective, validated measure of fatigue: the 5-item Modified Fatigue Impact Scale (MFIS-5) (6, 74, 85). Results indicated that STEPS strongly correlate not only with ambulatory function (6) but also with *worse MFIS-5 scores (r* = −0.44, *p* < 0.05).

##### Remote Monitoring Captures Fatiguability and State Fatigue

Initial research using bilateral foot-worn sensors (small IMUs) demonstrated the ability of spatio-temporal gait parameters to predict fatigue level (using the BORG scale for perceived exertion as a proxy for **state fatigue**) (86). Results from the foot-worn sensors demonstrate a significant change in gait parameters pre and post a 6-min walk test – providing information about the subjects' performance/fatigability. These data highlight the promising use of remote monitors as objective measures to evaluate fatigue as well as fatigability in PwMS both inside and out of the clinic setting.

Trait and state fatigue has been correlated to poor sleep quality and quantity (87). Increased physical activity (moderate-to-vigorous physical activity) has been correlated with improved sleep quality and reductions in subjective fatigue (88–90). Given the heterogeneity of symptoms associated with fatigue and the lack of insight into sleep quality and quantity in the home setting, remote devices monitoring sleep and physical activity are beneficial for evaluating personalized correlations on a patient-by-patient basis. Similarly, restless leg syndrome (RLS) is common in PwMS and has been correlated with higher fatigue (trait) and worse sleep quality and quantity (91)– using wearables to evaluate night-time lower extremity movement (from RLS) and sleep metrics can provide tailored information about factors exacerbating or involved in MS fatigue and potentially also fatiguability.

### General Limitations and Possible Solutions (i.e., Future Work)

Fatigue, by definition, remains a subjective symptom, and similar to pain, the measurement and monitoring tools will rely on patients' reports. Although subjective fatigue contributes to reduced physical, cognitive, and psychosocial activities among patients, many other factors result in decreased activity and fatigability. The pathophysiology of MS fatigue is also multifactorial and is different among patients and even for a given patient over the disease course. So, finding a single serological, cerebrospinal fluid, structural, or functional imaging biomarker for MS fatigue may not be attainable.

In this situation, we recommend combining ecological momentary fatigue assessment (i.e., for state and trait fatigue, using repeated questionnaires via smartphone applications) and remote real-world measurement of physical and cognitive function (fatigability) as a solution to this complex problem. Perhaps, it is possible to design a combined ‘composite score' that incorporates both subjective fatigue and objective fatigability into a single construct. Isolating the concept of fatigue from similar concepts, such as depression and excessive daytime sleepiness, and understanding how they affect and interact with each other may lead to more specific and targeted treatments for patients.

Looking forward, remote monitors can be used for therapeutic intervention. Exercise, as well as energy conservation methods, are known to be beneficial for treating MS fatigue (89, 92, 93). Using monitors can help personalize when, how and how much activity a person can perform before getting exhausted. A real-world example, from the FITriMS study, was the use of the Fitbit step count as a “dose-meter” – allowing the participant to know when they needed to slow down to ensure sufficient energy for the rest of the day, and even subsequent days.

## Conclusion

Subjective fatigue is one of the most common MS symptoms. Validated questionnaires are the most common tools for monitoring and measuring this disabling symptom. Most fatigue questionnaires can be administered remotely and can therefore be used for remote evaluation of fatigue in patients. Through deployment via smartphones and other mobile technologies, ecological momentary assessment may enable clinicians and researchers to better understand the patients' fatigue level, and its fluctuation and response to treatment in real-life settings. Objective decline of patients' function with exertion: what has been defined as fatigability, can be evaluated using wearable devices assessing level of physical activity - that can be influenced by fatigue severity. Wearables can also quantify the objective decline. By combining validated questionnaires, momentary and frequent subjective assessments, and objective measures of function and its decline with exertion, remote monitoring techniques will provide a more comprehensive picture of a patient's burden of symptoms and treatment response.

## Author Contributions

VB and BN contributed to conception and design as well as drafting and revision of the manuscript. RB contributed to concept and revision of the manuscript. All authors contributed to the article and approved the submitted version.

## Conflict of Interest

As recipient of the Career Transition Award, VB received funding from The National Multiple Sclerosis Society. As recipient of the Harry Weaver Award, RB received funding from The National Multiple Sclerosis Society. BN has received research funding from NMSS, PCORI, NIH, DoD and Gentech. BN also received personal fees from Jazz Pharmaceutical. The authors declare that the research was conducted in the absence of any commercial or financial relationships that could be construed as a potential conflict of interest.

## Publisher's Note

All claims expressed in this article are solely those of the authors and do not necessarily represent those of their affiliated organizations, or those of the publisher, the editors and the reviewers. Any product that may be evaluated in this article, or claim that may be made by its manufacturer, is not guaranteed or endorsed by the publisher.
